# Epidemiology, clinical characteristics, and associated cost of acute poisoning: a retrospective study

**DOI:** 10.1080/20523211.2024.2325513

**Published:** 2024-05-10

**Authors:** Waleed Salem, Pallivalappila Abdulrouf, Binny Thomas, Wessam Elkassem, Dina Abushanab, Haseebur Rahman Khan, Yolande Hanssens, Rajvir Singh, Hany A. Zaki, Aftab Mohammed Azad, Moza Al Hail, Shaban Mohammed

**Affiliations:** a Medical Toxicology, Trauma & Emergency Center, Hamad Medical Corporation, Doha, Qatar; b Drug and Poison Information Center, Pharmacy Executive Director’s Office, Hamad Medical Corporation, Doha, Qatar; c Department of Biostatistics, Heart Hospital, Hamad Medical Corporation, Doha, Qatar; d Medical Toxicology, Corporate Department of Emergency Medicine, Hamad Medical Corporation, Doha, Qatar

**Keywords:** Epidemiology, acute poisoning, emergency department (ED), cost, Qatar

## Abstract

**Introduction:**

Poisoning is a major public health issue and a leading cause of admission to the emergency department (ED). There is a paucity of data describing the epidemiology and cost of acute poisoning. Therefore, this study investigated the epidemiology, patterns, and associated costs of acute poisoning in emergency department of the largest tertiary care healthcare centre in Qatar.

**Method:**

This study was a retrospective review of the health records of patients admitted to the ED due to poisoning between January 2015 and December 2019. Incidence, clinical characteristics, and costs associated with acute poisoning were assessed. Frequency and percentages were calculated for categorical variables and mean and SD for continuous variables. The relationship between sociodemographic characteristics and poisoning profile was assessed using the chi-square test. A micro-costing approach using the cost of each resource was applied for cost calculations.

**Result:**

The incidence of acute poisoning was 178 cases per 100,000 patients. Females (56%) and children below 14 years (44.3%) accounted for the largest proportion. Most of the exposures were accidental involving therapeutic agents (64.2%). The mean length of hospital stay was found to be 1.84 ± 0.81 days, and most patients (76.6%) were discharged within the first 8 h. A statistically significant difference was found between age groups and type of toxin (χ2 = 23.3, *p* < 0.001), cause and route of exposure (χ2 = 42.2, *p* < 0.001), and length of hospital stay (χ2 = 113.16, *p* < 0.001). Admission to intensive care units had the highest cost expenditure (USD 326,008), while general wards accounted for the least (USD 57,709).

**Conclusion:**

Unintentional poisoning by pharmacological agents is common in infants and children. This study will assist in the development of educational and preventive programmes to minimise exposure to toxic agents. Further studies are required to explore the impact of medical toxicology services, and post discharge monitoring of poisoning.

## Introduction

Acute poisoning is an important public health concern and a leading cause of admission to emergency departments worldwide (Bulut et al., 2022; Rajabali et al. 2023). The World Health Organization (WHO) defines acute poisoning as ‘an illness caused by exposure to a toxic substance, which can occur through ingestion, inhalation, injection, or absorption through the skin, and has a rapid onset of symptoms and signs (World Health Organization, 2004). More than 83 million natural and synthetic substances have been registered in the Chemical Abstracts Service (CAS) Registry, of which less than 3000 substances are associated with more than 95% of all poisoning cases (Chemical Abstracts Service, 2014). Timely identification and diagnosis of toxins are vital for the management of acute poisoning. Furthermore, the prevalence and pattern of acute poisoning are dynamic and vary according to age, type of poisoning, intention, and geographical location (Abdollahi et al., 1997; Al-Jahdali et al., 2004). Therefore, country-specific data on the epidemiology and patterns of poisoning are imperative to address this issue and to minimise morbidity and mortality.

### Epidemiology and economic impact

According to a report published by the WHO, poisoning cases account for half a million deaths annually, with a loss of 6.3 million years of healthy life (disability-adjusted life years) (World Health Organization, 2004). In 2016, an estimated 800,000 people worldwide died of suicide alone (10.5 per 100,000 population), and most of these deaths occurred in low-to middle-income countries (Mittal et al., 2021). Data from the National Poison Data System (NPDS) collated and reported by the American Association of Poison Control Centers for the period January 2009 to January 2020 illustrate that acute poisoning is a major cause of morbidity and mortality in the US, affecting more than 2.2 million individuals annually where approximately 1.5 million are children (Gummin et al., 2021). A systematic review exploring the pattern and epidemiology of poisoning in Ethiopia revealed a high prevalence of poisoning among individuals below 30 years of age. The overall mortality ranged from 0 - 14.8%, and substance abuse and psychiatric problems were identified as the most common causes of acute poisoning (Chelkeba et al., 2018).

Epidemiological data suggest that an estimated 45,000 deaths due to poisoning occur annually among children and adolescents aged < 20 years (1.8 per 100,000 population). The majority of these cases were reported among children under five years of age, mostly due to medications administered incorrectly by the parents (Branche et al., 2008). In a further report (2020 -21) by the United Kingdom’s National Poisons Information Service, poisoning cases accounted for 170,000 hospital admissions, causing 4561 deaths (i.e. 79.5 deaths per million), of which 66% were associated with drug misuse, attributed mostly due to opiates and cocaine toxicity (Public Health England, 2022). A prospective observational study from Qatar exploring the clinical characteristics and determinants of poison cases admitted to the emergency department reported an incidence of 35.3/100,000 patients and a case-mortality rate of 0.39/1000. Most cases were accidental poisoning and occurred in females between 18 and 34 years of age. The most common toxic agents were chemicals, including household/cleaning materials, followed by pharmaceutical agents (Elmoheen et al., 2020).

While it is difficult to estimate the magnitude of the economic burden associated with poisoning, the cost associated with the opioid crisis has been estimated to be over $2.5 trillion (about $7,700 per person) from the United States alone (Marks, 2020). Rajabali et.al., examined the health and economic costs of poisoning in Canada, and the findings highlighted, out of 45,000 poison cases reported in 2016, nearly 10% led to hospital admissions and approximately 3% resulted in deaths at an annual cost of up to $812.5 million in the form of direct and indirect expenses. These findings further recommend targeted poisoning prevention programmes to reduce economic costs and long-term health consequences (Rajabali et al., 2023).

### Clinical characteristics and causes

The clinical presentation of acute poisoning varies depending on the substance or type of toxin, dose, duration, and concentration of the exposure. The most reported symptoms include gastrointestinal (nausea, vomiting, pain, diarrhea, etc.), respiratory (tachypnea), neurological (confusion, dizziness), and cardiac (bradycardia and dysrhythmias) symptoms (Lee et al., 2008). Poisoning occurs predominantly due to ingestion; however, injection, inhalation, or exposure of body surfaces also contributes to acute poisoning (Lapatto-Reiniluoto et al., 1998; Muhammad et al., 2018). Poisoning can be intentional or unintentional (accidental); while unintentional poisoning is more prevalent among children, deliberate use of toxic substances is associated with a high degree of morbidity and mortality (Al-Jahdali et al., 2004). While pharmaceutical products are a major cause of poisoning in developed countries, household insecticide chemicals are reported to be most frequently associated with poisoning in developing nations (Moradi et al., 2016).

### Rationale of the study

Despite significant adverse impacts on health, there is a dearth of global and regional data on the epidemiology, causes, and patterns of acute poisoning, and the existing data are not comparable largely due to differences in reporting systems, cultural and social factors, and variable access to healthcare services. In Qatar, studies on acute poisoning are scarce, and existing studies are limited to individual cases, pediatric populations, or studies originating from small settings (Khudair et al., 2013). Therefore, this study aimed to assess the epidemiology, clinical characteristics, outcomes, and overall costs associated with acute poisoning management in Qatar.

## Materials and methods

The study followed the Strengthening the Reporting of Observational Studies in Epidemiology (STROBE) guidelines to ensure high-quality presentations throughout the study (Von Elm et al., 2014).

### Study setting

The Hamad Medical Corporation (HMC) is the main provider of secondary and tertiary healthcare needs of approximately 2.9 million residents in Qatar. This study was conducted in the ED of the largest referral centre in Qatar, which provides 24/7 emergency medical services to patients of all ages. It is the main referral centre for poison and toxicology cases, and manages approximately 400,000 emergency cases annually. In 2017, a dedicated medical toxicology consulting service (MTCS) was established to treat poisoning and toxicology cases. The MTCS is a multidisciplinary team of medical toxicologists, pharmacists, and nurses who support the management of acute poisoning, drug overdoses, chemical exposure, and envenomation (Elmoheen et al., 2020).

### Study design & sampling framework

This was a retrospective, cross-sectional, observational study of all individuals presenting to the emergency department and diagnosed with acute poisoning between January 1, 2015, and December 31, 2019.

We used a systematic random sampling technique (Etikan & Bala, 2017; Singh & Masuku, 2014), and the 7th patient was randomly selected as a starting point, and the profile of every 10^th^ patient (17th, 27th, 37th etc.) was included. Systematic random sampling technique is efficient when dealing with larger samples as it provides representative subset of the population. Two independent reviewers (toxicologist and pharmacist) examined the relevance and completion of all the medical records.

### Data source

Data were obtained from electronic medical records based on the diagnoses made by the attending emergency physician. All patients who presented to the emergency department were either examined and treated by the emergency medical team or referred to MTCS. Depending on the severity of symptoms, patients were either treated and discharged from the emergency department or admitted to the intensive care unit/high dependency unit or other medical wards for further management.

### Data collection

Data abstraction was adapted from previous studies (Desalew et al., 2011; Mathew et al., 2019; Tefera & Teferi, 2020; Wakushie & Daba, 2016; Woyessa & Palanichamy, 2020). The poison cases were diagnosed based on the history given by the patients/family members, clinical presentation, and laboratory investigations. The data collection period was June to December 2021. Two research assistants extracted the data from the electronic medical records (Cerner) and the HMC toxicology service database (in-house database) and transferred them into an Excel data sheet. The resources consumed and their patterns of use in the management of poisoned patients were extracted from the medical records. Data collection included antidote costs, management, decontamination, laboratory and diagnostic tests ordered during hospitalisation, supportive measures taken, and length of hospital stay by ward type (ED, ICU, general medical ward, or admitted to a psychiatric hospital).

Antidotes included intravenous (IV) administration of N-acetylcysteine, hydroxocobalamin, naloxone, and atropine, whereas decontamination management included activated charcoal and gastric lavage. Laboratory tests included serum levels of acetaminophen, carbamazepine, phenytoin, salicylates, cholinesterase enzyme, lithium, tricyclic antidepressants, and valproic acid, while electrocardiography (ECG) was in the diagnostic test category. Intravenous fluids, oxygen, and mechanical ventilation were categorised as supportive treatment.

The cost of antidotes was collected from the pharmacy department and calculated based on the duration of therapy for each regimen from initiation to discontinuation of therapy. The costs associated with diagnostic tests were obtained from the HMC’s finance department (unit cost of each resource based on hospital charges) and included the cost of beds per patient, excluding other resources. All costs were based on the fiscal year 2022–2023, using the Qatari health Consumer Price Index (Trading Economics, 2023) and were presented in Qatari Riyal (QAR) and United States Dollar (USD). The costs of items not available in the HMC were obtained from published sources . A contemporary cost-adaptation approach, which was previously published and validated using health expenditure per capita and purchasing power parities, was also utilised (Ademi et al., 2018).

### Data analysis

The Statistical Package for Social Sciences (SPSS) version 29 (IBM Corp., Armonk, NY) was used for data analysis. Descriptive statistics and calculated frequency and percentages for qualitative variables and continuous variables were presented using mean and SD, and the relationship between sociodemographic characteristics and poisoning profile was assessed using the chi-square test. *P*-value less than 0.5 (two-tailed) was considered statistically significant level. The cost associated with admission was calculated and the total length of stay was calculated by summing the length of stay in each ward. In this study, a micro-costing approach was followed for the cost calculations using the unit costs of each resource. The total cost of management was calculated by multiplying the length of hospital stay in each ward by the total cost of the resources consumed in each ward. The Post-discharge costs were not included in this study.

## Results

### Incidence of acute poisoning

A total of 4006 out of 9325 cases were obtained from electronic medical records from January 2015 to December 2019. Of these, 401 were selected using systematic random sampling. Four cases were further excluded owing to incomplete data, leaving 397 unique cases that met the inclusion criteria. A total of 2,250,000 patients visited the ED during the study period. Therefore, the incidence of ED visits associated with poisoning was 178 per 100,000 patients.

### Demographics and characteristics of intoxicated patients

Descriptive information, including sex, age, nationality, occupation, cause of exposure, type of toxin, route, and outcome, is summarised in Table 1.

**Table 1.** Frequency distribution of demographic and clinical characters in the studied patients (*n* = 397).

Variable	Categories	Number of cases (n)	Percentage (%)
Gender	Male	173	43.6
	Female	224	56.4
Age group (years)	Infants (<1 year)	91	22.9
	Children (1–14 years)	176	44.3
	Adults (> 14 years)	130	32.7
Nationality	Qatari	130	32.7
	Non – Qatari	267	67.3
Psychiatric illness	Yes	17	4.3
	No	380	95.7
Occupation	Employed	74	18.6
	Unemployed	26	6.5
	Student	53	13.4
	Unknown	244	61.5
Toxicologist consulted	Yes	91	22.9
	No	306	79.6
Cause of exposure	Intentional	81	20.4
	Unintentional	316	77.8
Type of toxin	Pharmaceuticals	255	64.2
	Pesticides/gases and household items	68	17.1
	Envenomation/bites	56	13.9
	Others	18	4.8
Duration of stay	< 8 h	150	37.8
	1–2 days	175	44.1
	3–5 days	55	13.9
	> 5 days	17	4.3
Route	Oral Ingestion	315	79.3
	Bite (animals/insects)	56	14.1
	Inhalation (nasal)	22	11.8
	Parenteral	4	1
Outcome	Discharged within 24 h (includes stay in ED & short stay unit)	304	76.6
	Required inpatient admission for further management (HDU, ICU, etc.)	91	22.9
	Death	2	0.5
Abbreviations: HDU – High Dependency Unit, ICU – Intensive Care Unit			

Out of 397 cases, 56.4% were females and 43.6% were males, the mean age of all patients was 13.67 ± 16.9 with Inter quartile range of 4 (2–24.5) years. The youngest patient was less than one year old, and the oldest was 74-years old. Children aged between 1–14 years (44.3%) were most vulnerable to intoxication, followed by adults ‘above 14 years ‘of age (32.7%) and infants ‘below one year’ (22.9%). Accidental or unintentional poisoning (77.8%) was the major cause of exposure to toxins. Approximately one third of the patients were Qatari nationals (32.7%), and majority (95.7%) did not have previous history of psychiatric illness.

### Pattern of poisoning and type of toxins

Three hundred and sixteen (316/397; 77.8%) cases were related to unintentional poisoning (accidental) and 81 (20.4%) were identified as intentional poisoning. The cause of exposure varied by age, and there was a statistically significant difference between the different age groups and the manner of exposure to toxins (χ2 = 165.93, *p* < 0.001). Unintentional poisoning was most observed among children (53%), followed by infants (28.8%), and adults (17.4%). However, intentional poisoning was significantly higher among adults (92.6%) in comparison to children (7.4%) and infants (0). There was a statistically significant variance in the manner of exposure to the different types of toxic agents (χ2 = 20.09, *p* < 0.001). The data did not show any significant differences in the cause of exposure and factors such as sex, nationality, toxicology consultation, and treatment outcomes.

Table 2 summarises the different types of toxins used. Pharmaceutical drugs (64.2%) were reported to be the most frequent cause of acute poisoning. Multiple medications (19.1%), followed by antipyretics or analgesics (14.1%) and antihypertensive medications (8.1%) were the most frequently reported therapeutic agents. The ‘pesticides/household items/cleaning materials’ category (17.1%) was the second most common cause of poisoning, followed by envenomation (14.1%) due to insect and animal bites. In terms of bites, ant bites (7.56%) were most frequent, notably in children. Cleaning solutions (7.30%), pesticides (3.78%), and household items (3.78%), such as sodium hypochlorite (bleach), organophosphate, naphthalene, kerosene, also contributed to acute poisoning among different age groups.

**Table 2.** Different types of toxins and management.

Variables	Categories	Number (n)	Percentage (%)
Pharmaceutical agents (*n* = 255; 64.2%)	Multiple medications	76	19.1
	Antipyretics and analgesics	56	14.1
	Antihypertensives	32	8.1
	Herbs, vitamins and minerals	23	5.8
	Antidepressants	17	4.3
	Unknown	6	1.5
	Other medications	45	11.3
Insect and animal bites (*n* = 56; 14.1%)	Ant bite	30	7.56
	Bee sting	2	0.50
	Insect bite	13	3.27
	Scorpion sting	10	2.52
	Snake	1	0.25
Pesticides/ chemicals and household items (*n* = 68; 17.1%)	Cleaning solution & disinfectants (Sodium hypochlorite/ hydrochloric acid/ chloroxylenol)	29	7.30
	Pesticide (Organophosphate/naphthalene/ammonium chloride/brodifacoum etc.,)	15	3.78
	Household materials (gasoline petrol/kerosene/henna)	15	3.78
	Gases & chemicals (e.g. carbon monoxide)	9	2.27
Miscellaneous	Others	18	4.53
Treatment	Conservative management and pharmaceuticals Decontamination	333 154	83.88 38.79
	Antidotes and antivenom	30	7.56
	Mechanical ventilation and dialysis	9	2.23

Table 3 shows the associations between the age categories and other independent factors. There was a statistically significant difference between the age groups (infants vs. children vs. adults) and toxin types (χ2 = 23.3, *p* < 0.001). Among various routes of exposure, ingestion (79.3%) was the most common, followed by insect or animal bites (14.1%), inhalation (11.8), and parenteral route (1%). The data identified significant differences between different age groups and routes of exposure, i.e. intoxication through ingestion or oral route was significantly higher among infants (97.8%) and children (78.4%) in comparison to adults (67.7%) (χ2 = 42.2, *p* < 0.001). Similarly, envenomation was significantly higher among children aged 1–14 years (57.1%) in comparison to infants (3.6%) or adults (39.3%) at *p*< 0.001) (Table 4).

**Table 3.** Age vs categories.

	Category	<1 year (Infants) N (%)	1–14 years (children) N (%)	>14 years (adult) N (%)	*P* value
Gender	Male	43 (47.3)	94 (53.4)	36 (27.7)	<0.001
	Female	48 (52.7)	82 (46.6)	94 (72.3)	
Toxicologist consultation	No	78 (85.7)	147 (83.5)	81 (62.3)	<0.001
	Yes	13 (14.3)	29 (16.5)	49 (37.7)	
Reason for exposure	Intentional	0 (0)	6 (3.4)	75 (57.7)	<0.001
	Unintentional	91 (100)	170 (96.6)	55 (42.3)	
Type of agents	Pharmaceuticals	67 (73.6)	114 (64.8)	74 (56.9)	<0.001
	Pesticides/gases/ chemicals	15 (16.5)	22 (12.5)	31 (23.8)	
	Insect/animal bite	2 (2.2)	32 (18.2)	22 (16.9)	
	Others	7 (7.7)	8 (4.5)	3 (2.3)	
Outcome	Discharged <24 h	82 (90.1)	166 (94.3)	56 (43.1)	<0.001
	Required prolonged admission	9 (9.9)	10 (5.7)	72 (55.5)	
	Death	0 (0)	0 (0)	2 (1.5)	
Route	Ingestion	89 (97.8)	138 (78.4)	88 (67.7)	<0.001
	Inhalation	0 (0)	5 (2.8)	17 (13.1)	
	Bite/sting	2 (2.2)	32 (8.2)	22 (16.9)	
	Parenteral	0 (0)	1 (0.5)	3 (2.3)

**Table 4.** Outcome vs categories.

	Category	Discharged <24-hours	Required admission	Death	*P* value
Gender	Female	170 (55.9)	53 (58.2)	1 (50)	>0.05
	Male	134 (44.1)	38 (41.8)	1 (50)	
Age	Infant < 1 year	82 (27.0)	9 (9.9)	0 (0)	<0.001
	Children (1-14 years)	166 (54.6)	10 (11.0)	0 (0)	
	Adults (> 14 years)	56 (18.4)	72 (79.1)	2 (100)	
Reason for exposure	Intentional	26 (8.6)	55 (60.4)	0 (0)	<0.001
	Unintentional	278 (91.4)	36 (39.6)	2 (100)	
Type of agents	Pharmaceuticals	203 (66.8)	52 (57.1)	0 (0)	<0.001
	Pesticides/gases/chemicals	36 (11.8)	30 (33.0)	2 (100)	
	Insect/animal bite	51 (16.8)	5 (5.5)	0 (0)	
	Others	4 (4.6)	4 (4.4)	0 (0)	
Route	Ingestion	244 (80.3)	71 (78.0)	0 (0)	<0.001
	Inhalation	8 (2.6)	12 (13.2)	2 (100)	
	Bite/sting	51 (16.8)	5 (5.5)	0 (0)	
	Parenteral	1 (0.3)	3 (3.3)	0 (0)	

### Length of stay, treatment, and outcomes

In terms of duration of hospital stay, the mean patient days was found to be 1.84 ± 0.81, majority (81.9%) of the patients were discharged within the first 48 h of the admission, out of which nearly half (37.8%) were discharged within first 8 h. There was a statistically significant difference between age groups and length of hospital stay (χ2 = 113.16, *p* < 0.001). Adult patients had significantly longer length of stay when compared to children and infants, furthermore, the patients who stayed for 5 days or more were predominantly adults (94%). Similarly, there was a statistically significant variance in treatment outcomes, type of toxin (χ2 = 35.3, *p* < 0.001), and route of intoxication (χ2 = 60.8, *p* < 0.001) (Table 3. It was noted that nearly one fourth (22.9%) of the patients required admission to either the intensive care unit, high dependency, or inpatient wards. In terms of management, almost 84% of the patients were treated conservatively with therapeutic agents, decontamination using activated charcoal was used in 38.7% of patients. Less than one tenth of patients (7.5%) were treated with antidotes and antivenoms. Only nine (2.2%) patients required mechanical ventilation and/or dialysis for the management of poisoning. Two deaths due to the accidental inhalation of carbon monoxide (CO) gas have been reported in adults.

### Cost

Patients admitted to the ICU had the highest cost, QAR 1,189,931 (USD 326,008), followed by ED patients with QAR 725,833 (USD 198,858), while non-ICU general ward patients and psychiatric patients contributed the least to QAR 210,639 (USD 57,709) and QAR 27,202 (USD 7,453), respectively. The cost associated with the laboratory and diagnostic orders was highest among ED patients (i.e. total costs QAR of 8,025 (USD 2,199)), while psychiatric patients had the lowest total costs with a QAR of 613 (USD 168). Additionally, the total cost of other supportive management was highest in the ED (QAR 5,920 (USD 1,622) compared to QAR 17 (USD 5), only in the psychiatric ward. The costs of ICU stay were higher than those in the other wards: QAR 1,187,131 (USD 325,241) vs. QAR 711,816 (USD 195,018), QAR 205,840 (USD 56,394), and QAR 26,560 (USD 7,277) in the ED, non-ICU, and psychiatric wards, respectively.

A detailed description of the total cost and average costs in all patients and in each clinical ward setting is shown in Table 5 (supplementary files) (Table 6).

**Table 5.** Total cost outcomes associated with patients.

Resource	All patients, QAR (USD)	ICU, QAR (USD)	Non-ICU general ward, QAR (USD)	ED, QAR (USD)	Psychiatry, QAR (USD)
Hospitalization	2,131,347 (583,931)	1,187,131 (325,241)	205,840 (56,394)	711,816 (195,018)	26,560 (7,277)
Laboratory and diagnostic tests	16,747 (4,588)	2,737 (750)	4,527 (1,240)	8,025 (2,199)	613 (168)
Antidote and decontamination	174 (48)	35 (10)	91 (25)	72 (20)	11 (3)
Other supportive management	6,607 (1,810)	27 (7)	180 (49)	5,920 (1,622)	17 (5)
Total	2,154,876 (590,377)	1,189,931 (326,008)	210,639 (57,709)	725,833 (198,858)	27,202 (7,453)

QAR, Qatari Riyal, USD, United States dollar, ICU: intensive care unit, ED: emergency, *1 QAR is 0.27 USD.

**Table 6.** Average cost outcomes associated with patients.

Resource	All patients, QAR (USD)	ICU, QAR (USD)	Non-ICU general ward, QAR (USD)	ED, QAR (USD)	Psychiatry, QAR (USD)
Hospitalization	5,369 (1,471)	74,196 (20,327)	3,431 (940)	2,342 (642)	3,794 (1,039)
Laboratory and diagnostic tests	710 (195)	671 (184)	795 (218)	661 (181)	408 (112)
Antidote and decontamination	0.5 (0.)	2 (0.5)	2 (0.5)	0.3 (0.08)	2 (0.5)
Other supportive management	21 (6)	3 (0.8)	3 (0.8)	25 (7)	3 (0.8)
Total	6,100 (1,671)	74,872 (20,513)	4,231 (1,159)	3,028 (830)	4,208 (1,153)

QAR, Qatari Riyal, USD, United States dollar, ICU: intensive care unit, ED: emergency *1 QAR is 0.27 USD.

## Discussion

### Statement of key findings

This retrospective review describes the epidemiology, clinical outcomes, patterns, and associated costs of poisoning among patients admitted to the ED with HMC over five-year period. ED admissions in Qatar are high; the incidence of ED encounters for acute poisoning is 178 per 100,000 patients. Unintentional or accidental poisoning due to medication misuse is the most common type of poisoning reported in infants and children. Oral ingestion of pharmaceutical agents (64.2%) was the most common cause of acute poisoning. Most patients were managed conservatively using intravenous fluids or treated with pharmaceutical agents; fewer than 3% of patients required invasive interventions, such as mechanical ventilation or hemodialysis. Intentional poisoning was significantly higher among adults (57.7%), and most of these patients had longer length of stay in the hospital. The mortality rate in our study was found to be less than 0.5%, and almost three quarters of the patients were treated and discharged within the first 24 h of admission. Patients admitted to the ICU and ED had the highest costs, accounting for approximately USD 326,008 and USD 198,858, respectively.

### Interpretation of the findings

Acute poisoning is a major public health concern that adversely affects patients of all ages. The current study demonstrated the annual ED visits associated with poisoning to be 178 patients per 100,000 patients, which was consistent with the annual poison rates reported in other studies (0.14–7.2%) (Agarwal et al., 2016; Hanssens et al., 2001; Lee et al., 2019; Zhang et al., 2018). Accurate poisoning data is difficult to interpret due to several reasons such as patients’ reluctance to seek medical help, may be referred from other hospitals and admitted to the ICU or patients with missed diagnosis or were uncategorised etc.

In terms of sex, our study found higher incidence of poisoning among females (56.4%) when compared to their counterparts. These findings were similar to those published earlier in China, Iran, and Saudi Arabia (Adinew, 2016; Adinew et al., 2017; Bacha & Tilahun, 2015; Chala et al., 2015; Teklemariam et al., 2016; Wakushie & Daba, 2016). In contrast, few studies have highlighted the male predominance in some countries (Celine & Antony, 2015; Malangu, 2008). No significant association was found between sex and the presence of psychiatric disorders, reason for poisoning exposure, type of agent consumed, or outcome variables.

The study further demonstrated that poisoning varied between different age groups, with children between 1–14 years of age being the most affected. Infants and children accounted for approximately 70% of all cases of intoxication, and the majority involved accidental ingestion of poison, an observation that is consistent with other studies (Bari et al., 2014; Nair & Revi, 2015; Zhao et al., 2009; Zhigang et al., 2007). We anticipate that the natural curiosity and exploratory behaviour found in this age group is the leading cause of unintentional exposure to toxic substances. Similarly, oral ingestion is the major route (**≈** 80%) of poisoning, notably among the children, and the data corroborate with the findings of other studies (Molla et al., 2022). This might be due to the ease of oral administration compared to other routes.

Children, especially those in the pre-school age group, tend to explore objects by placing them in the mouth, which leads to unintentional accidental ingestion of harmful substances, such as pesticides, medications, cleaning materials, or household toxic substances. Furthermore, improper storage and disposal of unused medications in the house may also contribute to unintentional poisoning in children Kheir et al., 2011). Preventing poisoning among this age group is a multifaceted approach that involves adequate childproofing measures, such as keeping high-risk items out of reach of children, placing safety locks, and using child-resistant containers for storage. Furthermore, providing awareness and education to parents and caretakers about different toxins and potential associated risks, and proper storage of medications will be beneficial.

Therapeutic drugs were the most frequently used toxic agents identified in our study, followed by pesticides and envenomation by insects and animals. A similar pattern has been observed in studies originating from both developed and developing countries (Lam et al., 2010; Lapatto-Reiniluoto et al., 1998; Parker-Cote et al., 2018). Consuming multiple medications (more than one medication), followed by analgesics, antipyretics, and hypertensive medications, were the most frequently associated therapeutic subgroups in our study. Unintentional poisoning due to analgesics and antipyretics may be explained by the ease of availability of over-the-counter medications at home.. Several studies have also highlighted the lack of knowledge about medication use and irrational use of medications as key factors associated with unintentional poisoning (Chelkeba et al., 2018; Zhang et al., 2018). It is also noteworthy that the incidence of alcohol intoxication and opioid poisoning was found to be low, which is in contrast to a study published elsewhere (Afzali et al., 2008; Ayatollahi et al., 2011; Farzaneh et al., 2016) Exposure to pesticides, household items, and gases was the second most common cause of poisoning found in our study. ‘Among the household items, cleaning solutions and disinfectants such as sodium hypochlorite are the most frequent causes of hospital admission. This finding resonates with those previously published by Qatar and other researchers.

Unintentional poisoning was the predominant cause of poisoning reported in our study, which contrasts with the findings of other studies (Bundotich & Gichuhi, 2015; Ramesha et al., 2009). High employment rates, better socioeconomic status, good living standards, and high-quality education and healthcare services in Qatar may have contributed to low intentional poisoning or suicide rates.

In line with the findings from international literature, carbon monoxide (CO) toxicity in our study was classified as accidental poisoning. Two cases of serious poisoning and subsequent death due to CO inhalation were reported in the present study. Indoor use of charcoal grills in poorly ventilated camping tents has been reported as the cause of this incident. CO toxicity is common in this region and is mostly attributed to the use of coal stoves, charcoal for grills, building fires, shower systems, and exhaust gas from automobiles (Long et al., 2021). Preventive measures, such as appropriate public education about potential CO hazards and risk factors, installation of CO alarm systems, and replacement of coal with natural gas, have been proven to minimise such incidents.

Achieving better performance in public healthcare systems requires an effective strategic alignment. HMC toxicology management protocols are designed to achieve optimum management and reduce overall healthcare costs. This is the first attempt to analyse the economic consequences of poisoned patients admitted to Qatar. For patients not admitted to ICU, the cost was reduced to QAR 210,639 (USD 57,709). However, in the ICU, the cost of managing poisoned patients has increased to QAR 1,189,931 (USD 326,008). The main driver of these differences was the hospitalisation cost.

The literature and their results are particularly not comparable to our results, given the variations in resource utilisation and the nature of healthcare systems across the world. However, several studies have evaluated the cost of poisoning in patients admitted to the ED. For instance, from a Spanish perspective, Munoz et al. showed that the total cost of hospital care was higher than ours with €1,825,263 over 2.5 years of follow-up, resulting in a permanent occupation of four beds per year and driven by resources consumed in the ED (Muñoz et al., 2016). Similar to our findings, where an increase in the length of stay in the ICU contributed to more than 50% of the total cost increase, the main cost drivers in the Descamps et al. study, which was based in Belgium, were the ICU stay with a median cost of $4,859 for patients treated in ICU (Descamps et al., 2020). Here, however, similar to our findings, the cost of medications accounted for less than 1% of patient costs. These findings are also in line with those by Descamps et al., where the total direct costs in Belgium were higher than in our study with €1,512,346, and hospitalisation including the ICU stay contributed to lower costs than ours with €997,481 (Descamps et al., 2019).

### Limitations

The main limitation of this study was its retrospective design, which may have contributed to the missing patient data. Because poison cases were identified based on the diagnosis classified by the treating physicians, some cases would have been misclassified and, therefore, missed. We anticipate few patients might have missed, as they may have been directly transferred to the ICU after being referred from another hospital. Furthermore, the study did not assess risk factors or include data related to morbidity and mortality post discharge; thus, the mortality rates presented in this study may have been underestimated. Another limitation is the external validity of our study, which is limited to a single centre. Despite being the main referral centre, the findings may not reflect the exact situation in this region, thereby limiting the generalizability of the results.

### Conclusion

Most patients presenting to the ED with acute poisoning are infants or children with accidental exposure to therapeutic agents. Pharmaceuticals, pesticides, and disinfectants are commonly used toxic agents. Very low mortality rates and rapid recovery within 1–2 days indicate the accuracy of the diagnosis and high-quality emergency medical services in Qatar. Prospective studies in larger multicenter populations targeting risk factors, impact of medical toxicologists, predictors of clinical outcomes, and post discharge monitoring are highly recommended. Preventive measures, including public health education on the rational use and safe storage of therapeutic agents, pesticides, and disinfectants, will be useful in minimising accidental poisoning, notably in the pediatric population.

## Data Availability

The datasets used and/or analysed during the current study are available from the corresponding author upon reasonable request.

## Author information

**Dr. Waleed Salem** is a Senior Consultant, Medical Toxicologist, Emergency and Trauma, Hamad Medical Corporation, Doha, Qatar

**Dr. Pallivalappila Abdulrouf**, Drug Information Services, Pharmacy Research Center, Pharmacy Executive Director’s Office, Hamad Medical Corporation, Doha, Qatar

**Dr. Binny Thomas**, Drug Information Services, Antimicrobial Stewardship Program, Pharmacy Executive Director’s Office, Hamad Medical Corporation, Doha, Qatar

**Dr. Wessam Elkassem**, Department of Medication Safety and Quality, Pharmacy Executive Director’s Office, Hamad Medical Corporation, Doha, Qatar

**Ms. Dina Abushanab**, Drug Information Services, Pharmacy Executive Director’s Office, Hamad Medical Corporation, Doha, Qatar

**Mr. Haseebur Rahman**, Drug Information Services, Pharmacy Executive Director’s Office, Hamad Medical Corporation, Doha, Qatar

**Ms. Yolande Hanssens** Drug Information Services, Pharmacy Executive Director’s Office, Hamad Medical Corporation, Doha, Qatar

**Dr. Rajvir Singh,** Consultant Biostatistician, Heart Hospital, Hamad Medical Corporation, Doha, Qatar

**Dr. Hany A Zaki**, Medical Toxicologist, Emergency and Trauma, Hamad Medical Corporation, Doha, Qatar

**Dr. Aftab Mohammed Azad**, A/Chairman and Senior Consultant, Medical Toxicologist, Emergency and Trauma, Hamad Medical Corporation, Doha, Qatar.

**Dr. Moza Al Hail**, Executive Director of Pharmacy and Chair of Drug and Poison Information Center, Hamad Medical Corporation, Doha, Qatar
